# Access to water, sanitation and hygiene (WASH) services and drinking water contamination risk levels in households of Bishoftu Town, Ethiopia: A cross‐sectional study

**DOI:** 10.1002/hsr2.1662

**Published:** 2023-10-31

**Authors:** Aderajew Mekonnen Girmay, Sisay Derso Mengesha, Daniel A. Dinssa, Zinabu Assefa Alemu, Bedasa Wagari, Mesaye G. Weldegebriel, Melaku G. Serte, Tsigereda A. Alemayehu, Moa Abate Kenea, Abel Weldetinsae, Kirubel T. Teklu, Ermias Alemayehu Adugna, Kaleab S. Awoke, Tesfaye L. Bedada, Waktole Gobena, Getnet Fikreslassie, Wendayehu Wube, Vivian Hoffmann, Masresha Tessema, Getachew Tollera

**Affiliations:** ^1^ Department of Nutrition and Environmental Health Research Directorate Ethiopian Public Health Institute Addis Ababa Ethiopia; ^2^ Ministry of Health Addis Ababa Ethiopia; ^3^ International Food Policy Research Institute Washington District of Columbia USA; ^4^ Department of Economics and School of Public Policy and Administration Carleton University Ottawa Ontario Canada

**Keywords:** access, contamination risk level, hygiene, sanitation, WASH, water

## Abstract

**Background and Aims:**

Access to safe drinking water, sanitation, and hygiene is a fundamental human right and essential to control infectious diseases. However, many countries, including Ethiopia, do not have adequate data to report on basic water, sanitation, and hygiene (WASH) services. Although contaminated drinking water spreads diseases like cholera, diarrhea, typhoid, and dysentery, studies on drinking water contamination risk levels in households are limited in Ethiopia. Therefore, closing this gap needs investigation.

**Methods:**

A community‐based cross‐sectional study was conducted. A total of 5350 households were included. A systematic, simple random sampling technique was used to select the participants. The information was gathered through in‐person interviews using a standardized questionnaire. Furthermore, 1070 drinking water samples were collected from household water storage.

**Results:**

This investigation revealed that 9.8%, 83.9%, and 4.9% of households used limited, basic, and safely managed drinking water services, respectively. Besides, 10.2%, 15.7% and 59.3% of households used safely managed, basic and limited sanitation services, respectively. Yet, 10.6% and 4.2% of households used unimproved sanitation facilities and open defecation practices. Also, 40.5% and 19.4% of households used limited and basic hygiene services. On the other hand, 40.1% of households lacked functional handwashing facilities. In this study, 12.1%, 26.3%, and 42% of households’ drinking water samples were positive for *Escherichia coli*, fecal coliforms, and total coliforms, respectively. Also, 5.1% and 4.5% of households’ drinking water samples had very high and high contamination risk levels for *E. coli*, respectively. We found that 2.5% and 11.5% of households and water distributors had unacceptable fluoride concentrations, respectively.

**Conclusion:**

The majority of households in Bishoftu town lack access to safely managed sanitation, drinking water, and basic hygiene services. Many households’ water samples had very high and high health risk levels. Hence, the government and partner organizations should implement water and sanitation safety plans.

## 1 INTRODUCTION

Goal six of the United Nations sustainable development goals (SDGs) commits the international community to ensuring appropriate sanitation facilities, clean water, and hygiene services for all.^1^, ^2^ To monitor progress toward this goal, the World Health Organisation (WHO) and United Nations Children's Fund/UNICEF Joint Monitoring Programme (JMP) developed the JMP ladders for water, sanitation, and hygiene services.^3^ According to the most recent data collected by these agencies, 46%, 26%, and 29% of the global population still lack access to safely managed sanitation, safely managed drinking water, and handwashing facilities in their homes, respectively.^4^ In Africa, the need is even more acute, with 73%, 61%, and 63% of people lacked access to safely managed sanitation, drinking water, and basic hygiene services.^4^ In Ethiopia, only 11% of inhabitants used safely managed drinking water services.^5^ According to the 2016 Ethiopian Demographic Health Survey (EDHS) report, 65.2% of households had access to basic water services,^6^ while only 25.9% and 38% of households had access to basic sanitation and basic hygiene services, respectively, and 19.7% had access to improved sanitation services.^7^ On the other hand, 17% of Ethiopian households practised open defecation.^8^

A key human right and the cornerstone of public health is having access to clean water, proper sanitation, and hygiene services.^9^, ^10^ However, to attain the 2030 target for safely managed drinking water, sanitation, and hygiene, the least developed countries, including Ethiopia, will need to increase the rate of progress toward these goals by 10, 15, and 7 times, respectively.^4^ Low access to safe WASH (water, sanitation and hygiene) services is responsible for a large share of infectious disease transmission in developing countries,^11^, ^12^ and the number of people who suffer from WASH‐related health problems remains vast.^13^, ^14^ Nearly 525,000 children per year die from diarrheal diseases linked to contaminated water consumption and poor sanitation, making it the most common cause of death in children.^15^ According to a recent study, diarrheal disease accounted for over two‐thirds of the total WASH‐attributable burden, with over one million deaths and 55 million disability‐adjusted life years. This included 384,000 deaths from poor hand hygiene, 564,000 from poor sanitation, and 505,000 diarrhea deaths caused by contaminated drinking water. Overall, poor WASH access was responsible for 69% of the burden of diarrheal illness.^16^ The risk of developing waterborne illnesses, including diarrhea, typhoid, hepatitis A, and cholera, is high for about a quarter of those who use untreated surface water.^17^ Therefore, protecting water against contamination and regularly monitoring water safety at the source and household level is vital to improving water quality and public health.^18^, ^19^ Substantial evidence shows that safe WASH services are fundamental to control contagious diseases.^20^, ^21^, ^22^ The fact that many countries, including Ethiopia, do not have adequate data to report on basic WASH services constitutes a major burden to effective expansion of these services. Further, studies on drinking water contamination in Ethiopian households are extremely limited. Therefore, this study aimed to provide detailed information on households’ access to WASH services and to fill a gap in scientific evidence. Finally, this study will be significant input and play an essential role in developing an appropriate program planning and strategy, providing baseline information to policymakers and indicating where we are.

## 2 METHODS

### 2.1 Study area

The investigation was conducted in Bishoftu town. Bishoftu is 44 km from the capital city of Addis Ababa.^23^, ^24^ The crater lakes and large swamp areas are part of Bishoftu town's dominant features and play an important role in the area's rapid economic and socioeconomic development. The city has nine urban and five rural lowest administration levels, and an estimated 171,227 inhabitants.^25^ Bishoftu Town Water Utility Office (BTWUO) is the authority responsible for providing water supply and sewage services to the town. According to the office's unpublished recent report, the town receives its water from 30 boreholes, with a total daily water production of 26,000 cubic meters. This report also indicates that the BTWUO disinfects drinking water, but this is not done regularly. A project titled “Hygiene and Environmental Health Services” is now ongoing in the town. The project's purpose is to improve WASH services through a learning city model. The Ministry of Health Ethiopia chose the region containing Bishoftu for this project based on a variety of criteria, including its significance as a tourist destination, proximity to Addis Ababa, and suitability for supervision by national government leaders and experts.

### 2.2 Design

A community‐based cross‐sectional and experimental investigation was carried out in 2022.

### 2.3 Inclusion criteria

Inhabitants aged 18 years and above were included in the study, and those with mental and severe illnesses were excluded.

### 2.4 Sample size and sampling procedure

For this study, the sample size was calculated using a single population proportion formula with the assumption of 95% confidence interval (*Z*), 2% acceptable margin of error (*d*), 2 design effect (i), and based on a previous study, the proportion of households having access to safe water supply (*P*) was assumed to be 42%.^26^

Then, using the formula $\frac{(Z\alpha /2)*P\left(1-P\right)*e}{d^{2}}$, $n=\frac{4\times 0.42\;(1-0.42)2}{0.02\times 0.02}=4864\;\text{households}.$

Adding 10% for the nonresponse rate, the required sample size for this study was 5350.

In this investigation, 214 Enumeration Areas (EAs) were selected by the Ethiopian Central Statistics Service. Each EA was delineated using the Q‐field software. Following demarcation, fresh lists of households were gathered from each EA. A systematic, simple random sampling technique was used to select 25 households from each EA. To gather 25 households from each chosen EA, one household was initially identified using a lottery approach and additional households were included by adding the sampling interval. In total, 5350 households were included in the sample. An EA covers an average of 183 households. Five households per EA were selected for water samples in accordance with the JMP sampling technique for drinking water quality.^9^ To obtain a sampling interval, the 25 interviewed households were divided by five. One of these households was then chosen using a lottery system and the sampling interval (five) was added to select the remaining four households. Finally, 1070 drinking water samples were collected from household water storage containers. To compare household drinking water quality with that of drinking water reservoirs and distributors, 26 samples were also taken from 26 operational drinking water reservoirs and distributors.

### 2.5 Data collection

We selected 48 health experts with a bachelor's in science and extensive experience with similar data collection techniques. Eight supervisors with master's degrees were also recruited. The supervisors and data collectors received 5 days of training. Written consent was received from every study participant. Data were collected from the selected household members and heads of households. The information was gathered through in‐person interviews using a standardized questionnaire. The survey questionnaire consisted of questions on indicators of sociodemographic data, household waste management practices, and JMP core questions for water, sanitation, and hygiene services. Open Data Kit (ODK) software was used to gather the data. Drinking water sample collectors wore disposable gloves. Water samples were taken directly from the water storage of the households. The drinking water samples were collected with heat‐sterilized 250 mL plastic bottles for bacteria and 1000 mL for chemical characteristics. The bottles were taken to the lab within 6 h and maintained there in a refrigerator at a temperature of 4°C until analysis.

### 2.6 Analysis

The data were checked for accuracy before being imported into Stata 16 for analysis. A descriptive analysis was conducted. Variables were created based on the JMP ladders for the WASH definitions. The variables constructed for drinking water service ladders were safely managed, basic, limited, unimproved, and surface water. Similarly, variables created for sanitation service ladders were safely managed basic, limited, unimproved, and open defecation. Additionally, variables created for hygiene service ladders were basic, limited, and no hygiene service. To calculate the household wealth index, a principal component analysis was conducted. In this investigation, wealth index scores were assigned to households based on their possession of various consumer goods, such as a television, bicycle, or automobile, as well as dwelling attributes like a source of drinking water, toilet facilities, and flooring materials, in line with the Ethiopian Demographic and Health Survey (EDHS) by using similar asset types.^6^

#### 2.6.1 Bacteriological analysis

The parameters tested during this study were *E. coli*, total coliforms, and fecal coliforms. Total coliforms are found in the soil, vegetation, and human and animal intestines. They indicate the general sanitation of the water supply system and the pipeline's treatment efficiency. They are not pathogenic and do not themselves necessarily cause disease. Still, testing for total coliforms indicates drinking water safety. Contamination with fecal coliforms and *E. coli* specifically are direct indicators of fecal contamination and thus the risk that diarrheal pathogens are present. Bacteriological testing of the drinking water was done using the membrane filtration technique. To retain the indicator bacteria, a 100 mL water sample was seeped through a membrane filter with a 0.45 µm. Then, filters were aseptically taken out of the membrane holder, put on eosine methylene blue (EMB) agars, and incubated for 24 h at 37°C and 44.5°C to isolate total coliforms and fecal coliforms, respectively. Total coliform bacteria, the non‐spore‐forming bacilli, Gram‐negative bacteria, were detected using fermented lactose with acid and gas production following a 24‐h incubation at 37°C. The number of colonies was enumerated with direct light or using a colony counter. Colonies showing pink‐red colonies after 24 h of incubation at 37°C were considered presumptive total coliforms and counted and recorded in a logbook or worksheet. Then, five colonies with pink‐red colors were transferred to brilliant green broth (Heimedia) with an inverted dirham tube. After 24 h, lactose fermentation with gas production was considered as confirmed total coliform counts. Furthermore, fecal coliforms or thermo‐tolerant coliforms, typical pink‐red colonies that grow at 37°C, were enumerated. In addition, five colonies were transferred into five tubes of *E. coli* broth with an inverted Durham tube and incubated at 48°C. After that, tubes with lactose fermentation and gas production within a maximum of 48 h were considered thermo‐tolerant coliforms. In addition, *E. coli* was enumerated using AKJima test protocols. The test tubes with confirmed coliform counts were then transferred to peptone water and, after 24 h, checked for Indole production using Kovac's reagent. The tubes with positive Indole tests were then screened using the IMVIC biochemical test, and finally, *E. coli* was enumerated.^27^

#### 2.6.2 Fluoride and nitrate analysis

Using an ion‐selective electrode (ISE), the amount of fluoride in the water sample was determined. To achieve a pH of 5.2–5.4, the optimal pH range for fluoride detection, a total ionic strength adjustment buffer (TISAB) was used. Fluoride standard solution (100 mg/L F^−^) was prepared by diluting 10.0 mL of METTLER TOLEDO ISE 1000 mg/L F^−^ stock solution to 100 mL with deionized water. The 100 mg/L fluoride standard was diluted to create standard fluoride solutions in the 0.5, 1, 5, 10, and 20 mg/L series for the calibration of ISE. To calibrate the Ion ion‐selective electrode, 5 mL of TISAB and 5 mL of each calibration solution were combined in a beaker. The device automatically adjusts the slope value to 59 mV, which is within the permitted range (54–60 mV). In a 50 mL plastic beaker, the same volume of TISAB (5 mL) and sample (5 mL) were combined, and the mixture was thoroughly agitated while being measured. Next, the fluoride potentiometer's reading of the concentration was taken. The Ultraviolet spectrophotometer screening method was also used to measure the nitrate concentration.^28^ To 50 mL of a filtered sample, 1 mL of 1 N HCL solution was added to prevent interferences from hydroxide or carbonate and mixed thoroughly. A set of NO_3_^−^ standards (2, 4, 6 and 8 mg/L) were created and handled the same way as samples to create the standard curve. Finally, transmittance was measured at wavelengths of 275 nm to assess interference caused by dissolved organic matter and 220 nm to obtain a reading for NO_3_^−^. By subtracting absorbance at 275 nm twice from the absorbance at 220 nm and plotting the standard calibration curve, the concentration of NO_3_^−^ was determined.

### 2.7 Ethical approval

The research proposal, which was given the reference number EPHI‐IRB‐358‐2021, was approved by the Ethiopian Public Health Institute's scientific and ethical review board. The participants’ written permission was acquired. Nobody was made to participate in the study if they did not want to. The responders’ information was treated uniformly and never made available to anybody else.

### 2.8 Applied international definitions

#### 2.8.1 Drinking water

According to the World Health Organization (WHO) drinking water guideline, water samples with <1 colony forming unit (CFU)/100 mL for *E. coli* were considered uncontaminated, and samples with ≥1 CFU/100 mL were contaminated.^29^ Additionally, drinking‐water samples with ≤1.5 and ≤50 mg/L concentration of fluoride and nitrate values were considered to be free from contamination, and samples with >1.5 and >50 mg/L concentration values of fluoride and nitrate were supposed to be contaminated, respectively. Besides, contamination risk levels for *E. coli* were calculated according to many studies.^29^, ^30^, ^31^ Based on this, drinking water samples were divided into four categories: 0 (no risk), 1–10 (low risk), 11–100 (high risk), and >100 CFU per 100 mL very high risk.

#### 2.8.2 Wealth index

The household wealth index is a measure of a family's overall standard of living.^32^

#### 2.8.3 JMP ladders for water, sanitation, and hygiene services in households

**Table:** 

Service level	Definition
Drinking‐water services	
Safely managed	Obtaining drinking water from an improved source that is available on‐site, on‐demand, and uncontaminated by feces or high‐priority chemicals (in our case, fluoride and nitrate).
Basic	Using an improved source of water as long as the round‐trip collection time is under 30 min, including queuing.
Limited	When collecting water from an improved source, the round‐trip time, including queuing, exceeds 30 min.
Unimproved	Drinking water from an unprotected spring or a dug well.
Surface water	Directly obtaining drinking water from a pond, lake, dam, stream, river, or irrigation canal.
Sanitation services	
Safely managed	Utilize improved sanitation facilities where excreta are safely disposed of on‐site or collected and treated offsite and are not shared with other households.
Basic	Use of improved facilities that are not shared with other households.
Limited	Use of improved facilities that are shared with other households.
Unimproved	Use of bucket latrines, hanging latrines, and pit latrines without a slab or platform.
Open defecation	Disposal of human excrement with other solids or in open bodies of water, shrubs, beaches, or other accessible areas.
Hygiene services	
Basic	Presence of a handwashing facility with soap and water at home.
Limited	Presence of a handwashing facility lacking soap and/or water at home.
No services	There is no handwashing station at home.

## 3 FINDINGS

### 3.1 Sociodemographic characteristics

For this investigation, 5350 households participated, with a 98.9% response rate. In the study, 18% of responders had no ability to read or write. Besides, 15.6% of households were in the poorest wealth group based on the asset index described above (Table 1).

**Table 1:** Sociodemographic characteristics of participants.

Study variables	Frequency	Percentage (%)
Sex		
Female	3212	60.7
Male	2079	39.3
Level of education		
No skill in reading and writing	952	18
Having the skill of reading and writing	387	7.3
Grades 1 to 12	2727	51.5
Vocational	472	8.9
University level	753	14.2
Family size of the respondents		
≥4 family members	1152	21.8
<4 family members	4139	78.2
The wealth index of the households		
Poorest	824	15.6
Poorer	978	18.5
Middle	1102	20.8
Richer	1173	22.3
Richest	1214	23

### 3.2 Water quality and contamination risk levels

In this investigation, 12.1%, 26.3%, and 42% of households’ water samples were positive for *E. coli*, fecal coliforms, and total coliforms, respectively. Also, 5.1% and 4.5% of households’ drinking water samples had very high and high contamination risk levels, respectively. The mean scores of *E. coli*, fecal coliforms, and total coliform colony forming units (CFU)/100 mL were 46, 106, and 206, respectively. We found that 2.5% and 11.5% of households’ and distributers’ drinking water samples had unacceptable fluoride concentrations, respectively. Of the 26 drinking water samples taken from water distributors and reservoirs, 3.8% and 7.7% were positive for fecal and total coliforms, respectively (Table 2).

**Table 2:** Water quality and contamination risk levels.

Indicators of contaminants (CFU/100 mL) and mg/L			Frequency	%	Mean
Drinking water source		Unimproved	78	1.5	
		Improved	5213	98.5	
Households’ drinking‐water contamination risk levels	Having total coliforms	Yes	449	42	206
		No	621	58	
	Having fecal coliforms	Yes	281	26.3	106
		No	789	73.7	
	Having *E. coli*	Yes	129	12.1	46
		No	941	87.9	
	Risk category and risk score (number of *E. coli* CFU/100 mL)	Low (if <1 count)	941	87.9	
		Moderate (if 1–10 count)	27	2.5	
		High (if 11–100 count)	48	4.5	
		Very high (>100 counts)	54	5.1	
	Fluoride concentration	Unacceptable (>1.5 mg/L)	27	2.5	0.8
		Acceptable (≤1.5 mg/L)	1,043	97.5	
	Nitrate	≤50 mg/L	1,070	100	3
Distributors and reservoirs’ drinking‐water contamination risk levels	Having total coliforms	No	24	92.3	23
		Yes	2	7.7	
	Having Fecal coliforms	No	25	96.2	23
		Yes	1	3.8	
	Having *E. coli*	No	26	100	0
	Fluoride	Unacceptable	3	11.5	0.9
		Acceptable	23	88.5	
	Nitrate	Acceptable	26	100	2.9
Households’ liquid waste management practice		Connected to sewer	180	3.4	
		Connected to septic tank	127	2.4	
		Connected soak/seepage pit	402	7.6	
		Into toilet facility	847	16	
		Open space outside the premises	1588	30	
		In the compound	1852	35	
		Watering the garden	245	4.6	
		To open a ditch or water body	47	0.9	
		Others	3	0.1	

### 3.3 Access to the new JMP ladders for drinking water services

**Figure 1:**
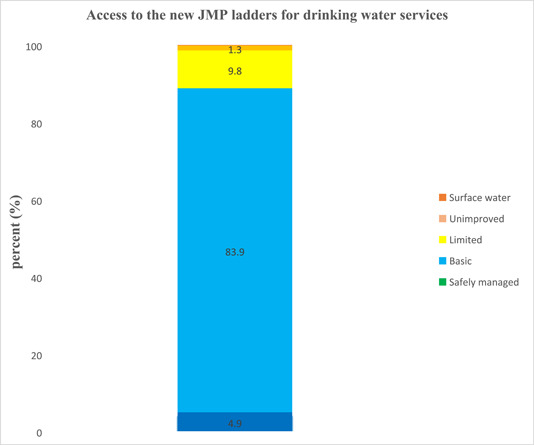
Access to the new Joint Monitoring Programme ladders for drinking water services.

In this investigation, 518 (9.8%), 4437 (83.9%), and 258 (4.9%) of households used limited, basic, and safely managed drinking water services. From the total households, 66 (1.3%) and 12 (0.2%) used unimproved and surface water, respectively (Figure 1).

### 3.4 Access to the new JMP ladders for sanitation services

**Figure 2:**
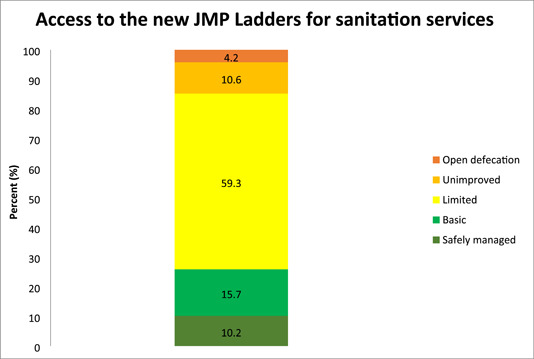
Access to the new Joint Monitoring Programme ladders for sanitation services.

In this survey, 829 (15.7%) and 542 (10.2%) of households used basic and safely managed sanitation services, respectively. Of the entire households, 3138 (59.3%) relied on limited sanitation services. In addition, 561 (10.6%) of households used unimproved sanitation facilities. Besides, 221 (4.2%) of households had open defecation practices (Figure 2).

### 3.5 Access to the new JMP ladders for hygiene services

**Figure 3:**
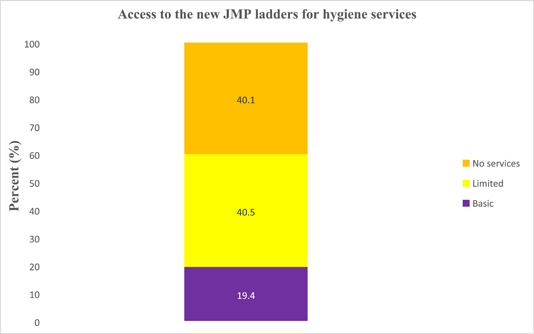
Access to the new Joint Monitoring Programme ladders for hygiene services.

In this investigation, only 1028 (19.4%) of households used basic hygiene services, meaning they had handwashing facilities with soap and water. Moreover, 2141 (40.5%) of households used limited hygiene services, meaning they had handwashing facilities lacking soap and/or water. However, 2122 (40.1%) of families had no functional handwashing facilities (Figure 3).

## 4 DISCUSSION

Attaining SDG 6 is vital to reduce many public health problems. However, in this investigation, access to safely managed sanitation facilities, drinking water, and basic hygiene services was low. Many households still lack access to clean water, adequate sanitation facilities, and hygiene services despite the 2030 Agenda for Sustainable Development's aim for universal access. Economic factors may be the primary cause since approximately 19% and 16% of households were determined to be the poorer and poorest, respectively. This idea was assisted by many studies.^33^, ^34^

Low access to WASH services implies that bold decisions by the country's political leaders are required to drive change at the rate and scale needed to meet the SDGs by 2030. In this study, only 4.9% of households had access to safely managed drinking water services, meaning access to improved sources of drinking water, accessible on‐premises, obtainable when required and uncontaminated by feces and high‐priority chemicals. This figure was less than Ethiopia's World Bank data from 2020, which showed that 12.6% of the inhabitants had access to safely managed drinking water services. The main reason could be using different microbial and chemical parameters. However, the current finding is consistent with the World Bank data of 2020 for the rural part of Ethiopia, where 5.3% of the population uses safely managed drinking water services. According to the JMP report of 2020, 25% of people in the world lacked safely managed drinking water services.^4^ Hence, coverage in developing countries, including Ethiopia, is expected to be low. This study revealed that 9.8% and 83.9% of households had access to limited and basic drinking water services, respectively.

On the other hand, 0.2% and 1.3% of households used surface water and unimproved drinking water sources, respectively. These households require immediate and urgent action to prevent waterborne diseases, particularly diarrheal diseases. In this study, 98.5% of households used improved drinking water sources. This was higher than the Ethiopian Demographic Health Survey (EDHS) 2016, where 97% of urban households used improved water sources.^6^ Also, this result was greater than an investigation performed in Northwest Ethiopia, where 78.9% of households used improved drinking water sources.^35^ This could be due to urbanization and economic differences. However, despite the high rate of improved water use, this study showed that 42% of samples of drinking water tested positive for total coliforms. This rate was greater than an investigation conducted in Tigray, Ethiopia.^36^ On the other hand, only 3.8% and 7.7% of the drinking water distributors and reservoirs were positive for fecal and total coliforms, respectively. *E. coli* was not found in reservoir or distributor drinking water samples.

The higher prevalence of total coliforms in household water thus appears to be due to poor household drinking water storage practices. In this study, 26.3% of the drinking water samples were contaminated with fecal coliforms. The main causes could be a lack of improved sanitation facilities and hand hygiene practices. Additionally, 30% of households used open space to manage their liquid waste, which could be the main reason for water contamination. Further, this study found that 12.1% of household drinking water samples tested positive for *E. coli*. Compared to research performed in Addis Ababa, this outcome was lower.^37^ The microbiological results of this study revealed that the drinking water management and effectiveness of the drinking water treatment practices were very low and could cause a significant public health problem.

The investigation revealed that 5.1% and 4.5% of households’ drinking water had very high and high contamination risk levels, respectively. These findings call for urgent and priority action to reduce public health problems. Regarding chemical parameters, 97.5% and 100% of households’ drinking water samples had acceptable fluoride and nitrate concentrations. Similarly, the study's findings revealed that 88.5% and 100% of distributor's and reservoirs’ drinking water samples had the WHO permissible and acceptable levels for fluoride and nitrate concentrations, respectively. In this study, 11.5% of distributor's and reservoirs’ drinking water samples had unacceptable or above the WHO permissible level (>1.5 mg/L) of fluoride concentration. The fluoride contamination may have several adverse health effects, primarily dental fluorosis or skeletal fluorosis, which can harm the bones and joints of the residents. However, only 2.5% of households’ drinking water samples had above‐permissible fluoride concentration levels. The difference can be due to precipitation, adsorption and ion exchange, membrane filtration processes, and the availability of drinking water treatments, including desalination.

The current study's findings revealed that only 10.2% of households had access to safely managed sanitation services. This rate was lower than the 2021 JPM report, where over 50% of global inhabitants had safely managed sanitation facilities.^4^ We found that above half (59.3%) of households used limited sanitation services. This demonstrated that many households used improved sanitation facilities that were shared with other households. This may expose the households to a variety of health problems. Expanding access to safe sanitation also has the potential to enhance dignity.

Moreover, 40.5% of households used limited hygiene services, meaning they had handwashing facilities that lacked soap and/or water. In addition, this investigation indicated that 40.1% of the families had no functional handwashing facilities. This is a critical gap to be addressed if SDG 6 is to be achieved in this population by 2030.

## 5 CONCLUSION

The new JMP higher service ladders for WASH services demand great emphasis and call for significant improvement. A significant number of households still use surface water and open defecation practices. Drinking water samples from a large number of homes were polluted chemically and especially bacteriologically. A large number of household samples exhibited a very high contamination risk level. Hence, there is a call to enhance WASH services to achieve excellent practice. The government and partner organizations should implement water and sanitation safety plans to improve WASH services and reduce public health problems. Also, the authors recommend that state authorities and partner organizations support local‐level water, sanitation, and hygiene plans and strategies. The government should also promulgate strong water safety, sanitation, and hygiene policies and strategies to increase WASH services.

### 5.1 Strengths and limitations of the study

The study employed a representative sample and a global study tool utilized by JMP to track WASH services and enable cross‐national comparisons. Physical characteristics of water have been used as a proxy for the quality of drinking water in other studies. However, this study solely evaluated the drinking water quality with respect to microbiological and priority chemical contaminants due to their priority and financial constraints. Therefore, one limitation of this study was not assessing the physical characteristics of drinking water. A second limitation is that the study did not test for the pathogenicity of *E. coli*, or for the presence of other pathogens.

## AUTHOR CONTRIBUTIONS

**Aderajew Mekonnen Girmay**: Conceptualization; data curation; formal analysis; funding acquisition; investigation; methodology; resources; software; supervision; validation; visualization; writing—original draft; writing—review & editing. **Sisay Derso Mengesha**: Conceptualization; data curation; funding acquisition; investigation; methodology; resources; supervision. **Daniel A. Dinssa**, **Zinabu Assefa Alemu**, **Bedasa Wagari**, **Mesaye G. Weldegebriel**, **Melaku G. Serte**, **Tsigereda A. Alemayehu**, and **Moa Abate Kenea**: Data curation; formal analysis; investigation; resources; supervision. **Abel Weldetinsae**: Data curation; investigation; methodology; resources; supervision. **Kirubel T. Teklu** and **Wendayehu Wube**: Data curation; investigation; resources; supervision. **Ermias Alemayehu Adugna**: Formal analysis; resources; software; visualization. **Kaleab S. Awoke**, **Tesfaye L. Bedada**, and **Waktole Gobena**: Data curation; formal analysis; investigation; resources. **Getnet Fikreslassie**: Data curation; formal analysis; investigation; resources; writing—review & editing. **Vivian Hoffmann**: Investigation; validation; visualization; writing—review & editing. **Masresha Tessema** and **Getachew Tollera**: Investigation; project administration; resources; supervision; validation; writing—review & editing.

## CONFLICT OF INTEREST STATEMENT

The authors declare no conflict of interest.

## TRANSPARENCY STATEMENT

The lead author Aderajew Mekonnen Girmay affirms that this manuscript is an honest, accurate, and transparent account of the study being reported; that no important aspects of the study have been omitted; and that any discrepancies from the study as planned (and, if relevant, registered) have been explained.

## Data Availability

The study's authors have read and approved the final version of the manuscript. In addition, the authors confirm that the data backup of the current study results is accessible within the article and/or its extra resources.
